# [^18^F]-JK-PSMA-7 PET/CT Under Androgen Deprivation Therapy in Advanced Prostate Cancer

**DOI:** 10.1007/s11307-020-01546-0

**Published:** 2020-10-01

**Authors:** Felix Dietlein, Peter Mueller, Carsten Kobe, Heike Endepols, Melanie Hohberg, Boris D. Zlatopolskiy, Philipp Krapf, Axel Heidenreich, Bernd Neumaier, Alexander Drzezga, Markus Dietlein

**Affiliations:** 1 Department of Nuclear Medicine, University Hospital of Cologne, Kerpener Str. 62, 50937 Cologne, Germany; 2 Department of Medical Oncology, Dana-Farber Cancer Institute, Harvard Medical School, Boston, USA; 3 Institute of Radiochemistry and Experimental Molecular Imaging, University Hospital of Cologne, Cologne, Germany; 4 Institute of Neuroscience and Medicine, INM-5: Nuclear Chemistry, Forschungszentrum Juelich GmbH, Julich, Germany; 5 Department of Urology, University Hospital of Cologne, Cologne, Germany; 6 Department of Urology, Medical University Vienna, Vienna, Austria

**Keywords:** Prostate cancer, Androgen deprivation therapy, PET, PSMA, [^18^F]-JK-PSMA-7

## Abstract

**Purpose:**

PSMA imaging is frequently used for monitoring of androgen deprivation therapy (ADT) in prostate cancer. In a previous study, [^18^F]-JK-PSMA-7 exhibited favorable properties for tumor localization after biochemical recurrence. In this retrospective study, we evaluated the performance of [^18^F]-JK-PSMA-7 under ADT.

**Procedures:**

We examined the performance of [^18^F]-JK-PSMA-7 in 70 patients (first cohort) with increasing or detectable PSA values under ADT (PSA < 2 ng/ml for 21/70 patients). We further analyzed 58 independent patients with PSA levels < 2 ng/ml under ADT, who were imaged with [^68^Ga]PSMA-11 or [^18^F]DCFPyL (second cohort). Finally, we compared detection rates between [^18^F]-JK-PSMA-7, [^68^Ga]PSMA-11, and [^18^F]DCFPyL.

**Results:**

In the first cohort, we detected [^18^F]-JK-PSMA-7-positive lesions in 63/70 patients. In patients with PSA levels ≥ 2 ng/ml, the detection rate was 100 % (49/49). In patients with PSA < 2 ng/ml, the detection rate was significantly lower (66.7 %, 14/21, *p* = 9.7 × 10^−5^) and dropped from 85.7 % (12/14, PSA levels between 0.3 and 2.0 ng/ml) to 28.6 % (2/7) for PSA levels < 0.3 ng/ml (*p* = 1.73 × 10^−2^). In the second cohort (PSA < 2 ng/ml), the detection rate was 79.3 % (46/58) for [^68^Ga]PSMA-11 or [^18^F]DCFPyL. Again, the detection rate was significantly higher (*p* = 1.1 × 10^−2^) for patients with PSA levels between 0.3 and 2.0 ng/ml (87.0 %, 40/46) relative to those with PSA levels < 0.3 ng/ml (50 %, 6/12). No significant difference was found between [^18^F]-JK-PSMA-7 and [^68^Ga]PSMA-11 or [^18^F]DCFPyL in patients with PSA levels < 2 ng/ml (*p* = 0.4295).

**Conclusion:**

[^18^F]-JK-PSMA-7 PET showed a high detection rate in patients with PSA levels ≥ 0.3 ng/ml under ADT. The lower PSA threshold of 0.3 ng/ml for high detection rates was consistent across the three PSMA ligands. Thus, PSMA imaging is suitable for clinical follow-up of patients with increasing PSA levels under ADT.

## Introduction

Several PSMA ligands are available for PET imaging in patients with prostate cancer, including [^68^Ga]PSMA-11, [^18^F]DCFPyL, and [^18^F]PSMA-1007 [1–4]. Recently, our group introduced the PSMA-specific derivative 2-MeO-[^18^F]DCFPyL for PET imaging, which is commonly referred to as [^18^F]-JK-PSMA-7 (J = Juelich; K = Köln) [5–7]. Like the other [^18^F]-labeled PSMA ligands, [^18^F]-JK-PSMA-7 is suited for high-throughput production in patient care.

PSMA PET is now included in international guidelines for imaging in biochemical recurrence (BCR) [8]. Additionally, PSMA PET scans are often in high demand for monitoring of patients with relapsed or metastasized prostate cancer. Under continuous androgen deprivation therapy (ADT) in castration-resistant prostate cancer (CRPC) with PSA values of > 2 ng/ml, Fendler and colleagues reported PSMA positivity in 196/200 patients [9]. These patients had mainly received [^68^Ga]PSMA-11 (*n* = 195) while some were examined with [^18^F]DCFPyL (*n* = 5). When administering enzalutamide or abiraterone in addition to an LHRH analog in CRPC, expression levels of PSMA increase in metastases, as recently shown in a cohort of 7 patients [10]. Thus, PSMA-specific PET tracers are not negatively affected by ADT once metastases have become castrate resistant.

In contrast to castrate-resistant patients, the impact of ADT on PSMA expression in hormone-sensitive prostate cancer (HSPC) remains unclear. A comparison of PET scans before and after therapy with ADT revealed a heterogeneous pattern of change in the PSMA expression of metastases in small cohorts of 10, 7, and 5 patients, respectively [10–12]. In the majority of these patients, PSMA expression in tumor metastases decreased or stopped completely after the start of ADT. To date, the influence of ADT on PSMA expression has been investigated primarily for the tracer [^68^Ga]PSMA-11.

Data on the probability of PSMA-positive lesions in patients with low PSA levels under continuous ADT are sparse. There is no consensus as to which PSA value for PMSA PET imaging is a sensitive tool under ADT. The definition of castration-resistant prostate cancer in the guidelines of the European Association of Urology includes a castrate serum testosterone < 50 ng/dl plus three consecutive rises in PSA, resulting in two 50 % increases over the nadir, and a PSA level > 2 ng/ml [8]. Hence, PSA levels of both < 2 ng/ml and > 2 ng/ml are among the criteria determining treatment plans.

Here, we report the results of the [^18^F]-JK-PSMA-7 PET/CT from the first year of clinical implementation in patients under ADT who were referred for PET imaging by their local urologists with a broad spectrum of PSA levels. The observed detection rate in patients with PSA levels < 2 ng/ml was re-examined in a second cohort investigated with [^68^Ga]PSMA-11 or [^18^F]DCFPyL.

## Materials and Methods

### Patient Characteristics and Study Design

#### Hypotheses and Study Design

We hypothesized that the detection rate of PSMA-positive metastases under continuous ADT was higher in patients with PSA levels ≥ 2 ng/ml relative to patients with PSA levels < 2 ng/ml (hypothesis 1). Our first cohort was imaged with [^18^F]-JK-PSMA-7 in 2017 as a routine clinical procedure (cohort 1). As stated in the “Results” section, hypothesis 1 was confirmed. Based on this finding, we next hypothesized that high detection rates could be achieved for patients with PSA levels < 2 ng/ml under ADT (hypothesis 2). To test this hypothesis, we retrospectively analyzed an independent cohort that consisted exclusively of patients with PSA levels < 2 ng/ml under ADT. These patients were imaged with [^68^Ga]PSMA-11 or [^18^F]DCFPyL as a routine clinical procedure in 2015 and 2016 (cohort 2). The advantage of cohort 2 was that more patients could be included in the group used to calculate the lower PSA threshold for beneficial imaging with PSMA agents. To determine the lower PSA threshold, we continuously adjusted the PSA threshold and divided the number of PSMA-positive patients with PSA levels above this threshold by the total number of patients with PSMA-positive lesions. Finally, we compared the detection rates for PSMA-positive lesions in patients with PSA < 2 ng/ml between cohorts 1 and 2, assuming the non-inferiority of the PSMA ligands [^18^F]-JK-PSMA-7, [^68^Ga]PSMA-11, and [^18^F]DCFPyL to one another.

In this IRB-approved study (20-1308), all patients (cohorts 1 and 2) gave their written, informed consent to PET imaging and inclusion of their data in a retrospective analysis. All procedures were carried out in compliance with the regulations of the local authorities responsible (District Administration of Cologne, Germany).

#### Cohort 1, All PSA Levels Under ADT, [^18^F]-JK-PSMA-7

The radiochemical development of the [^18^F]-JK-PSMA-7 tracer was completed in 2016 [5] and the non-inferiority of [^18^F]-JK-PSMA-7 in comparison with [^68^Ga]PSMA-11 was demonstrated in a pilot study and then for clinical use in BCR patients within the first year of [^18^F]-JK-PSMA-7 application [7]. In 2017, the first year of clinical application, a cohort of 162 prostate cancer patients received [^18^F]-JK-PSMA-7 for different indications, including staging, BCR, and therapy monitoring. Our study group comprised of 70 patients (aged 70.1 ± 5.5 years) who had been referred in 2017 for PET imaging with [^18^F]-JK-PSMA-7 (348 ± 55 MBq) under continuous ADT with LHRH analogs and/or bicalutamide. Nine patients also received enzalutamide or abiraterone, and 11 patients had been treated with docetaxel. As a PSA level > 2 ng/ml is one of several criteria to determine therapy decisions in advanced prostate cancer under ADT [8], we chose a PSA threshold of 2.0 ng/ml to differentiate between two patient groups under ADT. The PSA level was < 2 ng/ml in 21 patients (mean PSA 0.72 ± 0.52 ng/ml) and ≥ 2.0 ng/ml in 49 patients (158.53 ± 575.47 ng/ml). In the subcohort of 21 patients with PSA levels < 2.0 ng/ml, we retrospectively identified a subgroup of 7 patients with PSA levels < 0.3 ng/ml (PSA 0.11 ± 0.11 ng/ml). The PSA level in the remaining 14 patients was 1.03 ± 0.33 ng/ml. Data on the PSA nadir were available in 10 of the 21 patients in whom the PSA level was < 2.0 ng/ml: Four patients showed an increase in PSA level by a factor > 5, while in another 5, the PSA level increased by a factor of 2–5, and in one patient an increase of < 50 % was observed.

The distribution of PET findings for different ranges of PSA is reported in Table 1.

**Table 1:** Results of [^18^F]-JK-PSMA-7 PET/CT in 70 patients under ADT for advanced prostate cancer. The number of PSMA-positive or PSMA-negative patients is specified by the PSA level

[^18^F]-JK-PSMA-7 PET/CT under ADT in advanced prostate cancer	PSMA neg.	PSMA pos.	T+	N+	M+	T+ N+	T+ M+	N+ M+	T+ N+ M+
PSA < 0.3 ng/ml	5	2	1	1					
PSA ≥ 0.3 ng/ml, but < 2.0 ng/ml	2	12	1	3	1		2	2	3
PSA ≥ 2 ng/ml	0	49	3	4	11	2	1	18	10
Total of all patients	7	63	5	8	12	2	2	20	13

*ADT*, androgen deprivation therapy; *PSMA*, prostate-specific membrane antigen; *T+*, PSMA-positive tissue within the prostate fossa; *N+*, PSMA-positive lymph node; *M+*, PSMA-positive lesion in the bone, lung, or liver

#### Cohort 2, PSA Level < 2 ng/ml Under ADT, [^68^Ga]PSMA-11, or [^18^F]DCFPyL

In total, 155/1048 patients, who had undergone PSMA PET/CT imaging in 2015 and 2016, received continuous ADT. In this cohort, we identified 58 patients, who were 70.9 ± 6.8 years old and presented with PSA levels < 2 ng/ml under continuous ADT. One patient had received docetaxel. The average PSA value of these 58 patients was 0.94 ± 0.59 ng/ml under ADT. In 12 patients, PSA levels were < 0.3 ng/ml (average PSA 0.07 ± 0.09 ng/ml), and in the remaining 46 patients, PSA levels were between ≥ 0.3 and 2 ng/ml in (average PSA 1.16 ± 0.35 ng/ml). Data on PSA nadir were available for 25 patients. In 16 patients, PSA levels had increased 5-fold and in 9 patients, PSA levels had remained stable or increased by no more than 50 %. Gleason scores were available in 41 patients and were < 8 in 18 patients, = 8 in 12 patients, and > 8 in 11 patients.

The distribution of PET findings for different ranges of PSA is reported in Table 2.

**Table 2:** Results of [^68^Ga]-PSMA-11 or [^18^F]-DCFPyL PET/CT in 58 patients under ADT in advanced prostate cancer when the PSA level was < 2.0 ng/ml. The number of PSMA-positive or PSMA-negative patients is specified by the PSA level

[^68^Ga]-PSMA-11 or [^18^F]-DCFPyL PET/CT under ADT in advanced prostate cancer	PSMA neg.	PSMA pos.	T+	N+	M+	T+ N+	T+ M+	N+ M+	T+ N+ M+
PSA < 0.3 ng/ml	6	6		1	5				
PSA ≥ 0.3 ng/ml, but < 2.0 ng/ml	6	40	8	16	7	1	1	5	2
Total of all patients	12	46	8	17	12	1	1	5	2

*ADT*, androgen deprivation therapy; *PSMA*, prostate-specific membrane antigen; *T+*, PSMA-positive tissue within the prostate fossa; *N+*, PSMA-positive lymph node; *M+*, PSMA-positive lesion in the bone, lung, or liver

#### Comparison of the PSMA ligands

The detection rate for PSMA-positive lesions in cohort 1, the subgroup with PSA < 2 ng/ml ([^18^F]-JK-PSMA-7) was compared with the detection rate in cohort 2 ([^68^Ga]PSMA-11 or [^18^F]DCFPyL) by a chi-square test.

### Imaging

PET imaging was performed as previously described [1–3, 6, 7]. In brief, all images were acquired on a Biograph mCT 128 Flow PET/CT scanner (Siemens Healthineers, Erlangen, Germany) and reconstructed using an ultra-high-definition algorithm. The same filters and acquisition times (flow motion bed speed of 1.5 mm/s) were used for the different PSMA ligands. PET imaging started from the middle of the thighs to the tip of the skull. PET imaging was started 2 h after the injection of [^18^F]-JK-PSMA-7 or [^18^F]DCFPyL and 1 h after the injection of [^68^Ga]PSMA-11. CT scans (slice thickness of 5.0 mm, pitch 1.2) were acquired in a low-dose technique with kV and mA modulation and adapted to the size of the patient. PET/CT scans were interpreted according to published criteria for standardization of PSMA PET/CT interpretation [13, 14] by a team of two specialists in nuclear medicine and one radiologist. Any disagreement was resolved in consensus. The same team interpreted PET/CT scans from cohorts 1 and 2.

### Tracer Synthesis

The detailed procedure for the radiosynthesis of [^18^F]-JK-PSMA-7 using the “minimalist light” protocol was described previously [5]. Two batches per week were produced at the Forschungszentrum Juelich in accordance with applicable good manufacturing practice (GMP). The GMP-based quality control measures included radiochemical purity, endotoxin testing, pH value, and the determination of residual content of solvents such as acetonitrile, acetone, tertiary butanol, and tetra-ethyl-ammonium-hydrogen-carbonate [TEAHC] [5–7].

## Results

### Detection Rate of [^18^F]-JK-PSMA-7 PET Under ADT (Cohort 1)

**Fig. 1.**
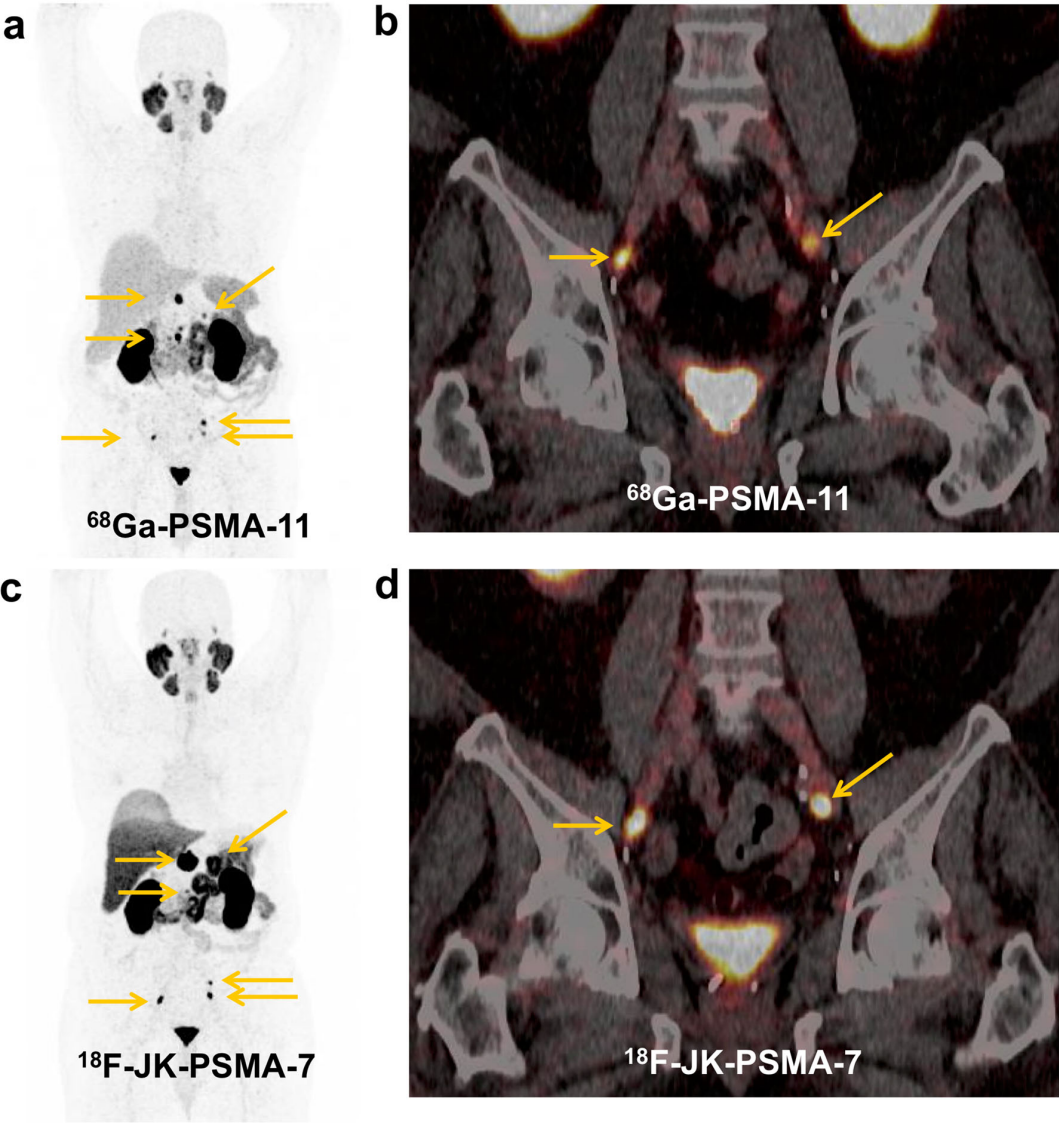
**a**, **b** Initial [^68^Ga]-PSMA-11 PET/CT (top) and **c**, **d** follow-up [^18^F]-JK-PSMA-7 PET/CT after 8 months (bottom). Concordant PSMA-positive lymph node metastases and PSMA-positive visceral metastasis in the left adrenal gland under ADT in a castrate-resistant patient with relatively low PSA levels. [^18^F]-JK-PSMA-7 PET shows progressive disease. During these 8 months, PSA levels increased from 0.2 ng/ml under ADT to 1.1 ng/ml under ADT.

**Fig. 2.**
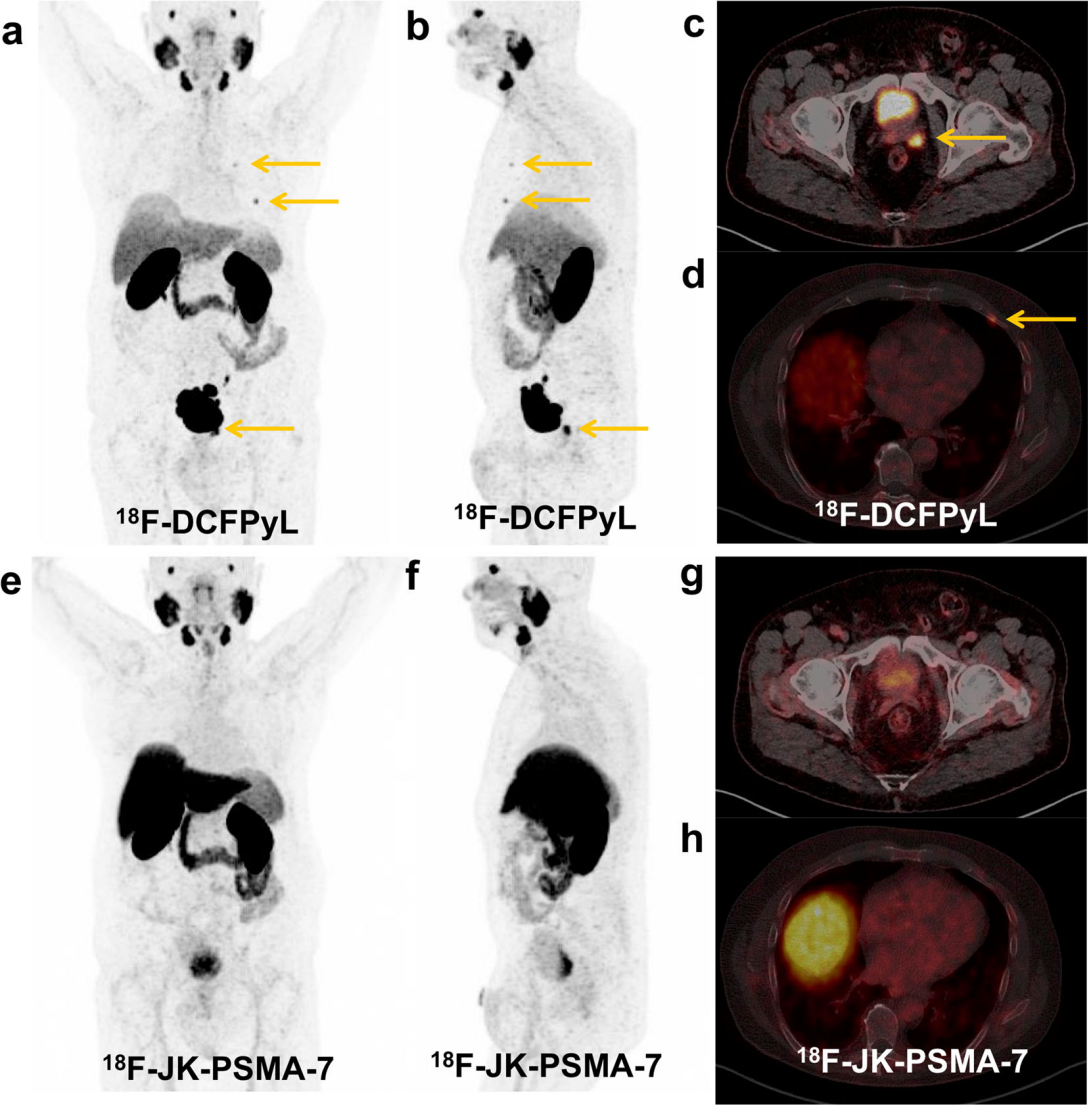
**a**, **b**, **c**, **d** [^18^F]-DCFPyL PET/CT (top) and **e**, **f**, **g**, **h** [^18^F]-JK-PSMA-7 PET/CT (bottom). The hormone-sensitive patient received irradiation due to the PSMA-positive relapse in the left seminal vesicle and ADT due to the PSMA-positive skeletal metastases in the 2nd and 4th ribs left. The [^18^F]-JK-PSMA-7 PET scan after 6 months became completely PSMA negative. The PSA level has dropped to 0.012 ng/ml.

**Fig. 3.**
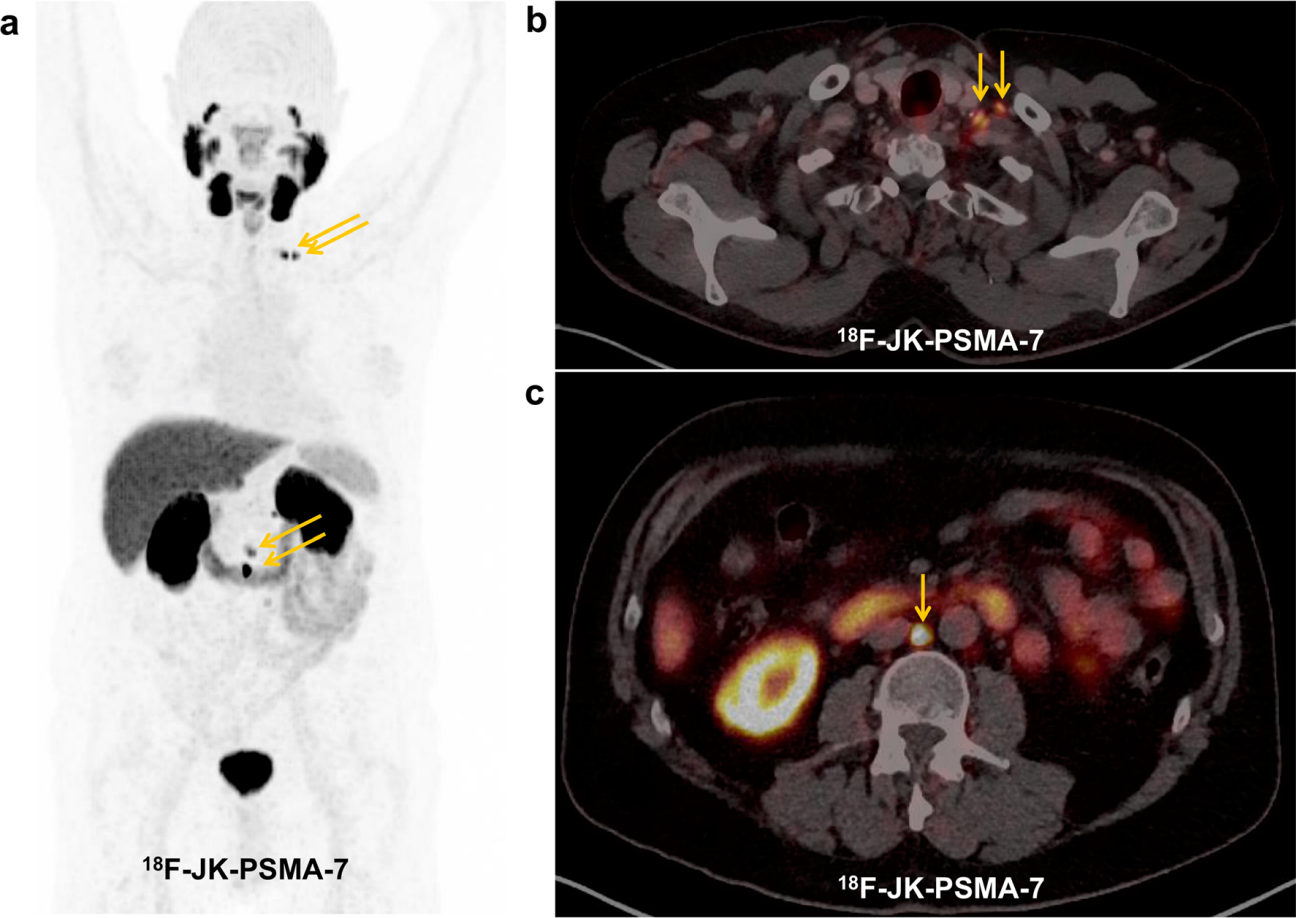
**a**, **b**, **c** The [^18^F]-JK-PSMA-7 PET/CT shows PSMA-positive lymph node metastases left cervical and retroperitoneal despite a low PSA level < 0.1 ng/ml under ADT.

We examined 70 prostate patients under continuous ADT with [^18^F]-JK-PSMA-7 (Table 1). Overall, we detected [^18^F]-JK-PSMA-7-positive lesions in 63/70 patients (90.0 %) (Figs. 1, 2, and 3). This detection rate varied with the PSA level under ADT and was significantly (*p* = 9.7 × 10^−5^, two-tailed Fisher’s exact test) higher in patients with PSA levels ≥ 2 ng/ml (100 %, 49/49) compared with PSA levels < 2 ng/ml (66.7 %, 14/21). However, individual patients with very low PSA levels still showed PSMA-positive lesions (Fig. 3).

**Fig. 4.**
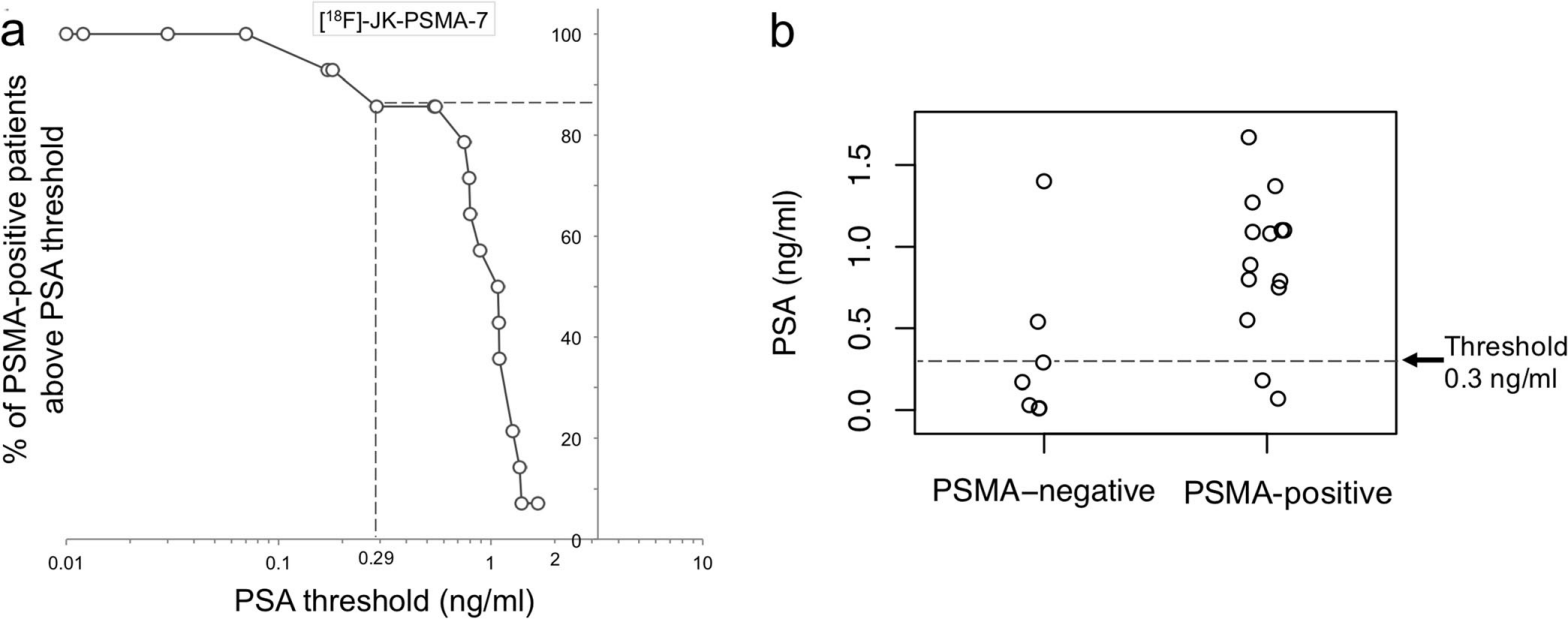
**a** We counted how many patients with PSMA-positive lesions had PSA levels above a given PSA threshold and divided this number by the total number of patients with PSMA-positive lesions. We then plotted the percentage of patients with a PSMA-positive [^18^F]-JK-PSMA-7 PET/CT (y-axis) against the PSA threshold (x-axis, log scale) for patients with PSA levels < 2 ng/ml. **b** Scattered dot plot of the distribution of [^18^F]-JK-PSMA-7 PSMA-negative and PSMA-positive PET/CT under ADT in patients with PSA < 2 ng/ml.

We therefore set out to derive a lower PSA threshold for imaging of prostate cancer patients under ADT that avoids unnecessary PSMA imaging but captures most PSMA-positive patients. In agreement with Ceci and colleagues [15], whose best nomogram-derived probability threshold was associated with a sensitivity of 84.7 %, we determined a PSA threshold for which ≥ 85 % of the patients with [^18^F]-JK-PSMA-7 positive will undergo PET imaging. Based on our subcohort of 21 patients with PSA levels < 2 ng/ml, 14 of these 21 patients showed PSMA-positive lesions, most of the PSMA-positive patients had PSA levels ≥ 0.3 ng/ml. For patients with PSA levels between 0.3 and 2 ng/ml under ADT, the detection rate with [^18^F]-JK-PSMA-7 was 85.7 % (12/14). This detection rate dropped significantly to 28.6 % (2/7) for PSA levels < 0.3 ng/ml (*p* < 1.73 × 10^−2^, two-tailed Fisher’s exact test) (Fig. 4a, b).

#### Verification

Sixty-three patients exhibited [^18^F]-JK-PSMA-7-positive lesions on the PET/CT scan. The majority (57/63) of these patients had a suspicious correlate on the CT scans. In 6 patients, PET scans were interpreted as [^18^F]-JK-PSMA-7 positive without any suspicious correlates on the corresponding low-dose CT scans. In these 6 patients, verification was based on an MRI scan (bone marrow infiltration, *n* = 1), on decreasing PSA levels after salvage radiotherapy of the prostate fossa or the pelvic lymph nodes (*n* = 3), or a follow-up PSMA PET/CT scan exhibiting tumor progression (*n* = 2).

### Detection Rates of [^68^Ga]PSMA-11 and [^18^F]DCFPyL Under ADT (Cohort 2)

**Fig. 5.**
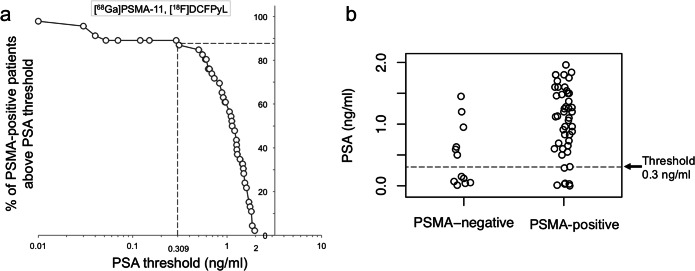
**a** The percentage of patients with a PSMA-positive [^68^Ga]-PSMA-11 or [^18^F]-DCFPyL PET/CT is plotted against the log-transformed PSA threshold for patients with PSA levels < 2 ng/ml under ADT. **b** Scattered dot plot of the distribution of pooled [^68^Ga]-PSMA-11 and [^18^F]-DCFPyL PSMA-negative and PSMA-positive PET/CT under ADT in patients with PSA < 2 ng/ml.

Based on our observations with [^18^F]-JK-PSMA-7, we hypothesized that many patients with PSA levels < 2.0 ng/ml under ADT exhibited robust expression of PSMA and that high detection rates could be achieved for patients with PSA levels < 2 ng/ml. To test this hypothesis, we examined the second cohort of 58 patients with PSA levels < 2 ng/ml under ADT who had undergone PET/CT imaging with [^68^Ga]PSMA-11 PET/CT (*n* = 50, 160.3 ± 35.1 MBq) or [^18^F]DCFPyL PET/CT (*n* = 8, 297.9 ± 73.6 MBq). In parallel to our previous analysis, we searched for a lower PSA threshold that included ≥ 85 % of the patients with [^68^Ga]PSMA-11-positive or [^18^F]DCFPyL-positive lesions (Fig. 5a, b). We found that 87.0 % (40/46) of the patients with PSMA-positive lesions had PSA levels ≥ 0.3 ng/ml under ADT, thus corroborating our previous observation. Overall, the detection rate was 79.3 % (46/58) and the detection rate differed significantly (*p* = 1.1 × 10^−2^, two-tailed Fisher’s exact test) between patients with PSA levels ≥ 0.3 ng/ml (87.0 %, 40/46) and < 0.3 ng/ml (50 %, 6/12) (Table 2). The 6 patients with PSMA expression despite PSA < 0.3 ng/ml had unfavorable Gleason scores (Gleason score < 8 in 1 patient, equal to 8 in 2 patients, and > 8 in 3 patients).

#### Verification

Forty-six patients showed [^68^Ga]PSMA-11-positive or [^18^F]DCFPyL-positive lesions. Most of these patients (38/46) had suspicious correlates on the corresponding CT scans. In 8 patients, PET scans were interpreted as PSMA positive without any suspicious correlates on the corresponding low-dose CT scans. In these 8 patients, verification was based on decreasing PSA levels after salvage radiotherapy of the prostate fossa or the pelvic lymph nodes (*n* = 3) or a follow-up PSMA PET/CT exhibiting tumor progression (*n* = 5).

### Comparison of the PSMA Ligands

The detection rates did not differ significantly between [^18^F]-JK-PSMA-7 from cohort 1 and [^68^Ga]PSMA-11 or [^18^F]DCFPyL from cohort 2 (*p* = 0.2453, chi-square test). Furthermore, no significant difference was found between [^18^F]-JK-PSMA-7 and [^68^Ga]PSMA-11 or [^18^F]DCFPyL, when the 3 PSMA ligands were analyzed separately (*p* = 0.4295, chi-square test).

## Discussion

This study revealed the following three findings on the PSMA imaging of prostate cancer under ADT:

- Using [^18^F]-JK-PSMA-7, PET/CT detection rates were statistically higher in prostate patients under ADT with PSA levels ≥ 2 ng/ml compared with patients with PSA level < 2 ng/ml.
- PSMA-positive lesions could be detected in many patients with PSA levels < 2 ng/ml under continuous ADT. Detection rates were high for PSA levels as low as 0.3 ng/ml. However, detection rates were significantly higher in patients with PSA levels ≥ 0.3 ng/ml than in patients with PSA levels < 0.3 ng/ml.
- Detection rates did not differ significantly between [^18^F]-JK-PSMA-7, [^68^Ga]PSMA-11, and [^18^F]DCFPyL in patients with PSA levels between 0.3 and 2 ng/ml. The lower PSA threshold of 0.3 ng/ml for high detection rates was consistent across the two cohorts: cohort 1 (using [^18^F]-JK-PSMA-7) and cohort 2 (using [^68^Ga]PSMA-11 or [^18^F]DCFPyL).

Our previous preclinical and clinical studies have demonstrated the favorable properties of the [^18^F]-JK-PSMA-7 ligand for tumor localization after biochemical recurrence and its robust synthesis [5, 7]. We now perform PSMA imaging frequently for monitoring of androgen deprivation therapy (ADT) in routine diagnostics. Substantially, fewer studies have examined the performance of PSMA imaging under ADT [9–11, 15] and our calculation of the lower PSA threshold for this clinical scenario is helpful for the accurate selection of patients under ADT for PSMA PET/CT imaging.

Consistent with a recent study by Fendler and colleagues [9], we found a robust overexpression of PSMA in the metastases of patients with PSA levels of ≥ 2 ng/ml under long-term ADT. Moreover, our study demonstrates that [^18^F]-JK-PSMA-7 PET has a high detection rate for patients under ADT with PSA levels as low as 0.3 ng/ml. This PSA threshold is as low as the thresholds derived from studies examining the performance of PSMA imaging based on biochemical recurrence after a curatively intended prostatectomy. Potential explanations of this observation include the possibility that tumor cells might increase their PSMA expression in response to long-term ADT.

An overview of PSMA PET studies under ADT is presented in Table 3. The largest cohort included 200 patients and demonstrated that the sensitivity of PSMA PET is high in CRPC under ADT [9]. Most patients in the largest cohort had PSA levels > 2 ng/ml (198/200). The present study demonstrates the potential of PSMA PET once PSA levels have exceeded 0.3 ng/ml under ADT. The association between detection rate and PSA level holds for [^18^F]-JK-PSMA-7, [^68^Ga]PSMA-11, and [^18^F]DCFPyL. However, ADT seems to have a negative impact on PSMA expression once ADT has started in hormone-sensitive patients, and a positive impact when enzalutamide or abiraterone has been added to long-term ADT in castrate-resistant patients. The pathophysiological model is that ADT upregulates the folate hydrolase 1 gene and thereby increases PSMA expression [20]. In clinical practice, PSMA imaging is feasible under long-term ADT as soon as the PSA level exceeds 0.3 ng/ml. Besides the PSA level, further predictors of a positive scan are PSA doubling time and grading [15, 20].

**Table 3:** Data on PSMA expression in different clinical settings under androgen deprivation therapy

Author	Patients	PSMA tracer	Results
Continuous ADT			
Fendler *et al*. [9]	200 pts., continuous ADT, nmCRPC, PSADT ≤ 10 month or Gleason score ≥ 8, PSA 1.3-263.8 ng/ml, median 5.3 ng/ml	[^68^Ga]-PSMA-11 (*n* = 195) or [^18^F]-DCFPyL (*n* = 5)	PSMA positive in 196/200 pts.
Ceci *et al*. [15]	61 pts; PSA progression under continuous ADT before second-line systemic therapies	[^68^Ga]-PSMA-11	PSMA positive in 86.9 %
Present study	70 pts. under continuous ADT, heterogeneous cohort, association between detection rate of PSMA-positive lesions, and PSA level	[^18^F]-JK-PSMA-7	PSMA expression in 49/49 of the pts. with PSA ≥ 2 ng/ml, in 12/14 of the pts. with PSA ≥ 0.3 ng/ml, but < 2 ng/ml, and in 2/7 of pts. with PSA < 0.3 ng/ml
Present study	58 pts. under continuous ADT, heterogeneous cohort, PSA level < 2 ng/ml	[^68^Ga]-PSMA-11 (*n* = 50) or [^18^F]-DCFPyL (*n* = 8)	PSMA expression in 40/46 pts. with PSA ≥ 0.3 ng/ml, but < 2 ng/ml, and in 6/12 pts. with PSA < 0.3 ng/ml
Start of ADT in HSPC			
Afshar-Oromieh *et al*. [11]	10 pts., start of ADT in HSPC	[^68^Ga]-PSMA-11 (baseline, days 42–369)	Heterogeneity of PSMA expression under ADT, PSMA negativity in 2/10 pts.
Leitsmann *et al*. [12]	5 pts., start of ADT in HSPC, decrease of the PSA level to 0.13–0.82 ng/ml	[^68^Ga]-PSMA-11 (baseline and days 6–11)	Heterogeneity of PSMA expression under ADT
Emmett *et al*. [10]	Cohort 1 (8 pts.): start of ADT in CSPC, cohort 2 (7 pts.): start of enzalutamide or abiraterone in CRPC	[^68^Ga]-PSMA-11 (baseline, day 8, day 18, day 28)	Cohort 1: heterogeneity of PSMA expression, cohort 2: increase of PSMA uptake on day 9, plateaued at day 18
Onal *et al.* [16]	108 pts., start of ADT in HSPC	[^68^Ga]-PSMA-11 (baseline, second scan after 2.9 months [median])	Decrease of PSMA expression in 91/108 pts. (91 %)
Ettala *et al*. [17]	9 pts., prospectively enrolled, start of degarelix (Firmagon®) in HSPC	[^68^Ga]-PSMA-11 (baseline and 3 times post-ADT)	Heterogeneous increase in PSMA uptake 3 to 4 weeks post-ADT
Start of enzalutamide or abiraterone in CRPC			
Emmett *et al*. [10]	Cohort 1 (8 pts.): start of ADT in CSPC, cohort 2 (7 pts.): start of enzalutamide or abiraterone in CRPC	[^68^Ga]-PSMA-11 (baseline, day 8, day 18, day 28)	Cohort 1: heterogeneity of PSMA expression, cohort 2: increase of PSMA uptake on day 9, plateaued at day 18
Wondergem *et al*. [18]	1 pt., start of enzalutamide in CRPC	[^18^F]-DCFPyL	Increase of PSMA uptake after 3 months
Rosar *et al*. [19]	10 pts., start of enzalutamide in mCRPC	[^68^Ga]-PSMA-11 (baseline, second scan after 11.8 day, [mean])	Increase of PSMA expression by a dimension of 50 %

*ADT*, androgen deprivation therapy; *CRPC*, castration-resistant prostate cancer; *HSPC*, hormone-sensitive prostate cancer; *mCRPC*, metastasized castration-resistant prostate cancer; *nmCRPC*, nonmetastasized castration-resistant prostate cancer; *PSADT*, PSA doubling time

The statistically calculated PSA threshold of 0.3 ng/ml under long-term ADT is conservative, as 42.1 % of the patients with PSA levels < 0.3 ng/ml had a PSMA-positive scan, when taking the results of the 3 PSMA ligands together. We found that in patients with unfavorable Gleason scores, PSMA PET can reveal PSMA-positive lesions even at lower PSA levels under ADT.

### Limitations

Our work demonstrates that [^18^F]-JK-PSMA-7 PET can detect PSMA expression in metastases and relapses under continuous ADT. Some patients were heavily pretreated. However, we have no information on castrate serum testosterone or on consecutive rises in PSA resulting in two 50 % increases over the nadir, which are defining criteria for hormone-sensitive or castration-resistant prostate cancer [8]. We therefore used the PSA level at the time of PET to differentiate between a low PSA level cohort and a high PSA level cohort. We further emphasize that we did not investigate PSMA expression on the commencement of ADT. On the other hand, the ENZAMET phase 3 trial has shown that the androgen-receptor inhibitor enzalutamide will improve survival in men with metastatic, hormone-sensitive prostate cancer [21]. In the future, a greater variety of therapeutic agents for ADT will be used as first-line therapy of patients with polytope metastatic prostate cancer.

Comparison of our [^18^F]-JK-PSMA-7 PET cohort with the independent [^68^Ga]PSMA-11 or [^18^F]DCFPyL PET cohort was hindered by the fact that we did not have details of the Gleason score in each patient and could not therefore carry out a matched-pair analysis.

## Conclusion

[^18^F]-JK-PSMA-7 PET imaging robustly detected PSMA expression when PSA levels exceed 0.3 ng/ml under continuous ADT. This result was reproducible for [^68^Ga]PSMA-11 and [^18^F]DCFPyL. Thus, PSMA PET is a suitable imaging tool even under ADT once the PSA level starts to rise.
